# Thermophysical Measurements on 90Ti-6AI-4V Alloy Above 1450 K Using a Transient (Subsecond) Technique*

**DOI:** 10.6028/jres.081A.014

**Published:** 1977-04-01

**Authors:** A. Cezairliyan, J. L. McClure, R. Taylor

**Affiliations:** Institute for Materials Research, National Bureau of Standards, Washington, D.C. 20234

**Keywords:** Electrical resistivity, heat capacity, high-speed measurements, high temperatures, melting, normal spectral emittance, radiance temperature, specific heat capacity, thermal radiation properties, thermodynamics, titanium alloy

## Abstract

Simultaneous measurements are described of specific heat capacity, electrical resistivity and hemispherical total emittance of the ternary alloy 90Ti-Al-4V in the temperature range 1450 to 1900 K, and the melting point and and the radiance temperature at the melting point of the alloy by a subsecond duration transient technique. The results are expressed by the relations:

$$\begin{array}{l} c_{p}=1.3833-9.943\times 10^{-4}T+3.745\times 10^{-7}T^{2}\\ \rho =152.65+1.9304\times 10^{-2}T-3.9548\times 10^{-6}T^{2} \end{array}$$

where *c_p_* is in J · g^−1^ · K^−1^, *ρ* is in *μ*Ω · cm, and *T* is in K. The value of the hemispherical total emittance is 0.39 in the range 1700 to 1900 K. The melting point and the radiance temperature at the melting point are 1943 and 1796 total emittance of the ternary alloy 90Ti-6Al-4V in the temperature range 1450 to 1900 K, and the melting point 0.395. Estimated inaccuracies of measured properties are: 3 percent for specific heat capacity, 1 percent for electrical resistivity, 5 percent for hemispherical total emittance and 8 K for melting point and radiance temperature at the melting point.

## 1. Introduction

In this paper, application of a transient technique to the measurement of selected thermophysical properties (specific heat capacity, electrical resistivity, hemispherical total emittance, melting point,^1^ radiance temperature^2^ at the melting point) of the alloy 90Ti-6A1-4V above 1450 K is described.

The method is based on rapid resistive self-heating of the specimen from room temperature to its melting point in less than one second by the passage of an electrical current pulse through it; and on measuring, with millisecond resolution, such experimental quantities as current through the specimen, potential drop across the specimen, and specimen temperature. Detailes regarding the construction and operation of the measurement system, the formulation of relations for properties, the methods of measuring experimental quantities, and other pertinent information are given in earlier publications [1, 2]^3^.

## 2. Measurements

### 2.1. Specimens

The measurements of specific heat capacity, electrical resistivity, hemispherical total emittance, and melting point were performed on four specimens in the form of tubes. The tubes were fabricated from rods by removing the center portion using an electro-erosion technique. The nominal dimensions of the specimens were: length: 76.2 mm; outside diameter, 6.3 mm; and wall thickness, 0.5 mm. A small rectangular hole (0.5 × 1 mm) fabricated in the wall at the middle of the specimen approximated blackbody conditions for optical temperature measurements. The outer surfaces of the specimens were polished to reduce heat loss due to thermal radiation.

The radiance temperature measurements at the melting point were performed on specimens in the form of strips fabricated from a plate. The nominal dimensions of the strips were: length, 51 mm; width, 4.7 mm, and thickness, 0.25 mm. Before the experiments, the surface of the specimens was treated using abrasive; three different grades of abrasive were used yielding three different surface roughnesses (ranging from approximately 0.4 to 0.9 *μ*m RMS) for different specimens.

**Figure 1:**
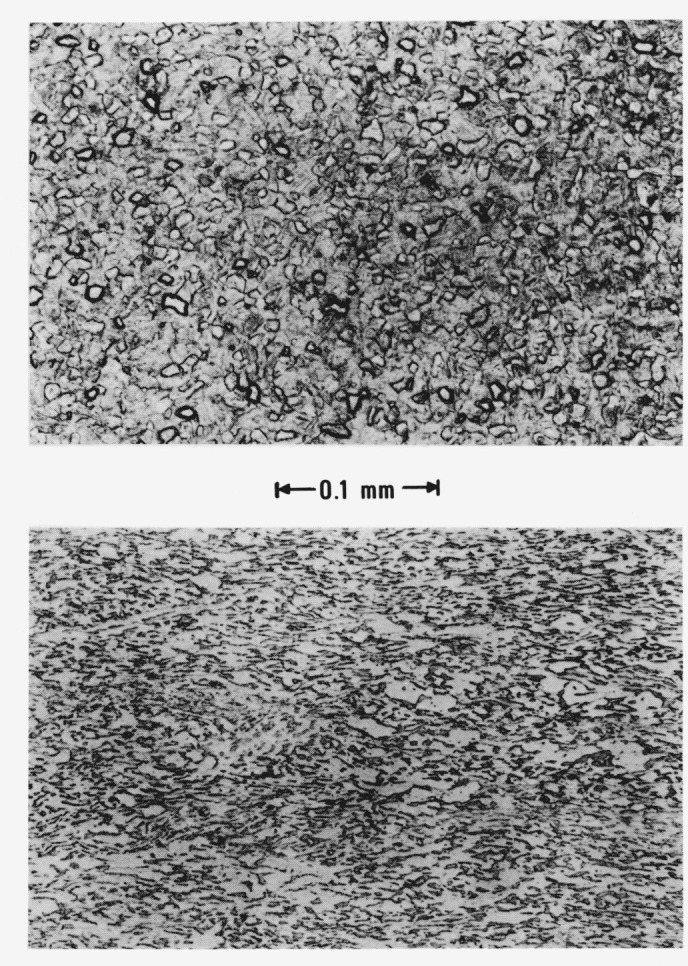
Photomicrographs of titanium alloy specimens: tube-shape (upper photograph), and strip-shape (lower photograph), obtained before the experiments.

The specimen material in rod and plate form was furnished by the U. S. Air Force Materials Laboratory, Wright Patterson Air Force Base, Ohio, and comprised two different lots of material. Chemical analyses of the material which are listed in table 1, show slight differences in composition. The rod material, from which the tube-shape specimens were fabricated, was hot swaged and the structure was composed of primary alpha titanium together with some acicular alpha + retained beta. The plate material, from which strip-shape specimens were fabricated, showed evidence of an elongated grain structure comprising primary alpha and beta. Photomicrographs of the two structures are shown in Figure 1.

### 2.2. Procedure

All the experiments were performed with the specimen in an argon environment at atmospheric pressure. To optimize the operation of the high-speed pyrometer, the temperature interval (1450 to 1943 K) was divided into three ranges. This yielded a total of twelve experiments on four tube-shape specimens. Duration of the current pulses in experiments on tube-shape specimens ranged from 400 to 500 ms, with heating rates ranging from 2600 to 3800 K·s^−1^. Duration of the current pulses in experiments on strip-shape specimens ranged from 400 to 800 ms, with heating rates ranging from 1300 to 2200 K·s^−1^. Radiative heat loss from the tube-shape specimens was, in all cases, less than 2 percent at 1500 K and less than 5 percent at 1900 K of the input power.

**Figure 2:**
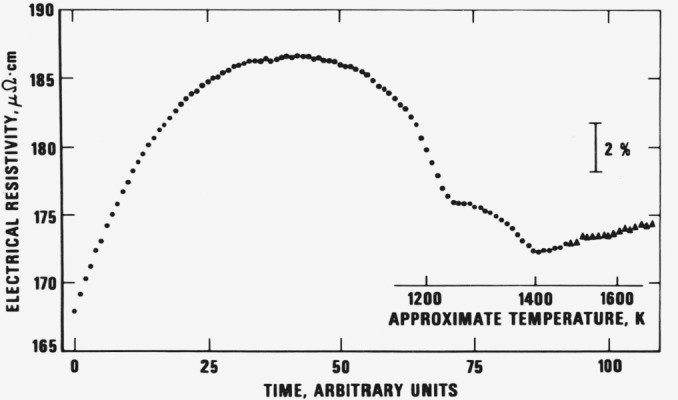
Variation of the electrical resistivity of the titanium alloy (specimen-2) as a function of time during its heating from near room temperature to 1700 K (1 time unit = 5 ms). The plotted points are the results of two consecutive experiments (dots: near room temperature to 1450 K, and triangles: 1450 to 1700 K). An approximate temperature scale (non-linear) is shown for the latter part of the heating.

At low temperatures and at vanadium contents greater than 3 percent, the equilibrium structure of this alloy comprises a mixture of the alpha and beta phases, although the actual structure will depend upon the heat treatment conditions. Above about 1300 K, the exact temperature depending upon composition, the alloy transforms to wholly beta. To ensure that the transformation was completed below 1450 K an additional experiment was performed in which the electrical resistance of a tube-shape specimen was measured during its heating from room temperature to the melting point (in 480 ms). The results, as shown in figure 2 indicate that the transformation occurred over a wide temperature range and that it was completed at about 1400 K, which is below the range of the results on thermophysical properties obtained in the present investigation.

## 3. Results

The thermophysical properties reported in this paper are based on the International Practical Temperature Scale of 1968 [3]. In all computations, the geometrical quantities are based on their room temperature (298 K) dimensions. The experimental results on specific heat capacity and electrical resistivity are represented by polynomial functions in temperature obtained by least squares approximation of the individual points. The final values on these properties, at 50 degree temperature intervals, computed using the functions are presented in table 2. The results obtained from individual experiments are given in the Appendix.

### Specific Heat Capacity

Specific heat capacity was computed from data taken during the heating period. A correction for power loss due to thermal radiation was made using the results on hemispherical total emittance. The function for specific heat capacity (standard deviation = 0.7%) that represents the results in the temperature range 1450 to 1900 K is:

$$c_{p}=1.3833-9.943\times 10^{-4}T+3.745\times 10^{-7}T^{2}$$ (1)

where *T* is in K, and *c_p_* is in J · g^−1^ · K^−1^.

### Electrical Resistivity

The electrical resistivity was determined from the same experiments that were used to calculate specific heat capacity. The function for electrical resistivity (standard deviation = 0.2%) that represents the results in the temperature range 1450 to 1900 K is:

$$\rho =152.65+1.9304\times 10^{-2}T-3.9548\times 10^{-6}T^{2}$$ (2)

where *T* is in K and *ρ* is in *μ*Ω · cm. In the computations of the specimen’s cross-sectional area, which is needed for the computations of electrical resistivity, the density of the specimen was taken as 4.422 g · cm^−3^. The measurement, before the pulse experiments, of the electrical resistivity of the four tube-shape specimens at 293 K with a Kelvin bridge yielded an average value of 166.2 *μ*Ω · cm with an average absolute deviation of 0.1 percent and a maximum absolute deviation of 0.2 percent.

### Hemispherical Total Emittance

Hemispherical total emittance was computed using data taken during both heating and initial free radiative cooling periods. The results of measurements in the temperature range 1700 to 1900 K did not show any significant variation in emittance. A value of 0.39 was obtained by averaging all the results (standard deviation = 2%).

### Melting Point

**Figure 3:**
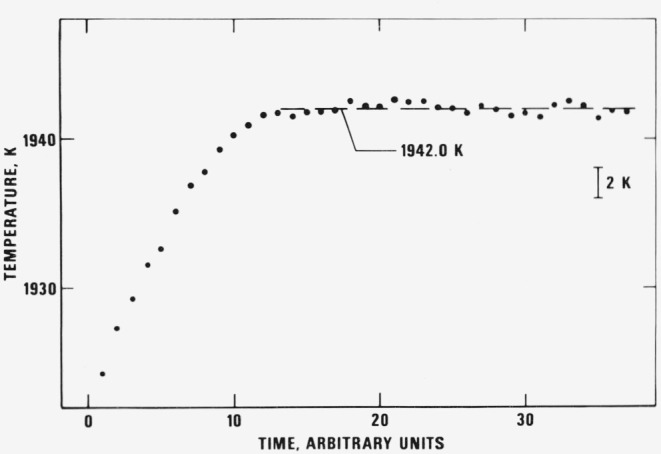
Variation of the temperature of the titanium alloy (specimen-3) as a function of time near and at its melting point (1 time unit = 0.833 ms).

Temperature of the tube-shape specimens was measured near and during the initial melting period until the specimen collapsed. A plateau in temperature indicated the transition from solid to liquid phase. Typical results for the variation of the specimen temperature during melting are shown in figure 3. The melting point for each specimen was obtained by averaging the temperature points on the plateau. The results are presented in table 3. The average melting point of the four specimens is 1942.6 K with an average absolute deviation from the mean of 0.4 K and a maximum absolute deviation of 0.8 K. It may be concluded that the melting point of the titanium alloy measured in this work is 1943 K.

### Radiance Temperature at the Melting Point

**Figure 4:**
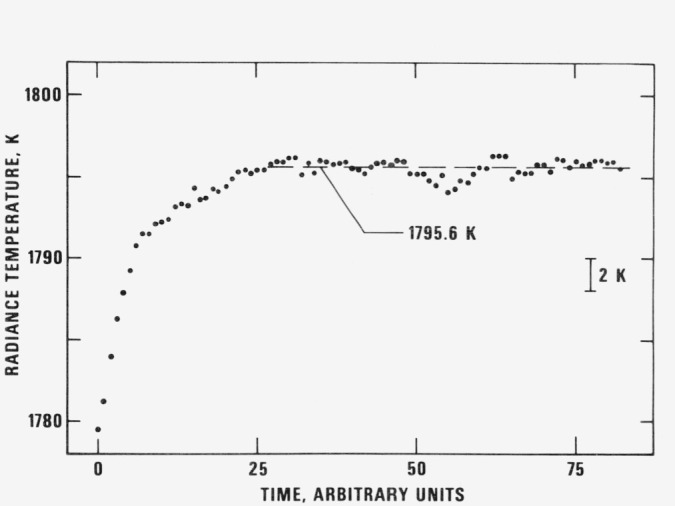
Variation of the radiance temperature (at 653 nm) of the titanium alloy (specimen-2) as a function of time near and at its melting point (1 time unit = 0.833 ms).

Radiance temperature measurements were performed on the strip-shape specimens at 653 nm which corresponds to the effective wavelength of the pyrometer’s interference filter. The bandwidth of the filter was 10 nm. The circular area viewed by the pyrometer was 0.2 mm in diameter. Typical results for the variation of the specimen radiance temperature during melting are shown in figure 4. A single value for the radiance temperature at the plateau was obtained by averaging the temperatures at the plateau. The results are presented in table 4. The average radiance temperature at the melting point for the specimens is 1795.6 K with an average absolute deviation of 0.2 K and a maximum absolute deviation of 0.4 K. It may be concluded that the radiance temperature of the titanium alloy measured in this work is 1796 K.

### Normal Spectral Emittance

The normal spectral emittance at the melting point was determined using the results of the radiance temperature (obtained from the measurements on strip-shape specimens) and the melting point (obtained from the measurements on tube-shape specimens). The results yield a value of 0.395 for the normal spectral emittance (at 653 nm) at the melting point of the alloy.

### Estimate of Errors

The details of estimating errors in measured and computed quantities using the present measurement system are given in an earlier publication [2]. In this paper, the specific items were recomputed whenever the present conditions differed from those in the earlier publication. The results are summarized in table 5.

## 4. Discussion

**Figure 5:**
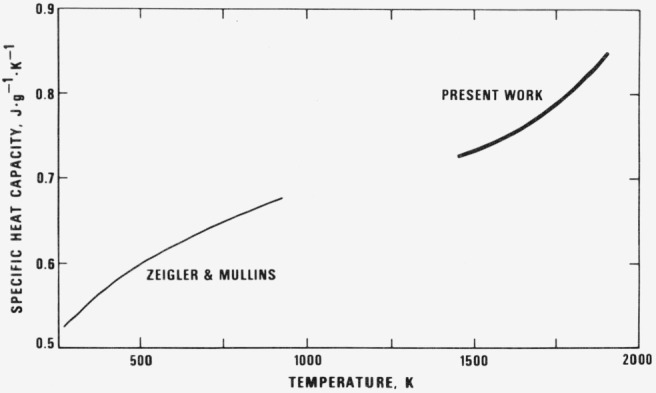
Specific heat capacity of titanium alloy reported in the literature. The alloy used by Zeigler and Mullins [4] contained 5.9 percent Al and 3.9 percent V.

**Figure 6:**
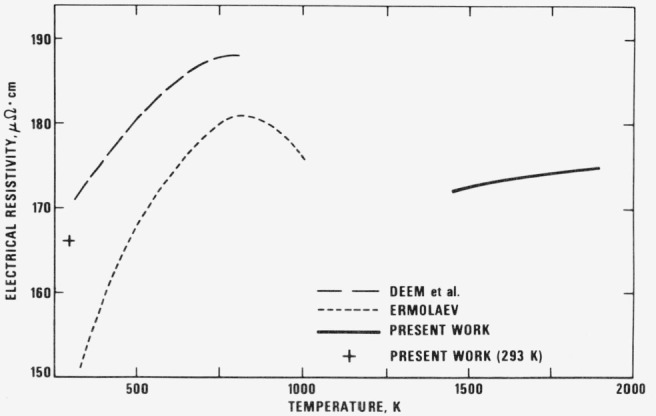
Electrical resistivity of titanium alloy reported in the literature. The alloy used by Deem et al. [5] contained 6 percent Al and 4 percent V, while that used by Ermolaev [6] contained 5 percent Al and 4 percent V.

Considering the wealth of literature data on 90Ti-6Al-4V there is remarkably little information on the physical properties of this alloy and data at temperatures above 1000 K does not appear to have been obtained. In figures 5 and 6 the smoothed values for specific heat capacity and electrical resistivity listed in table 2 are plotted together with the limited amount of low temperature data available in the literature. From figure 5, it can be seen that the specific heat capacity data of Ziegler and Mullins [4] may be reasonably extrapolated to join up with the present data.

The electrical resistivity data on 90Ti-6Al-4V reported by Deem et al. [5] covers the temperature range 310 to 810 K and shows evidence of a maximum around 800 K. Ermolaev [6] has measured the electrical resistivity of a titanium alloy containing 5 percent Al and 4 percent V up to 1000 K and his results are some 4 to 12 percent lower than the data of Deem et al. However, a definite maximum is observed around 800 K. These results, together with those of the present work, are presented in figure 6. The room temperature value obtained in the present work is about 2.5 percent below the value of Deem et al., but such a difference can be accounted for by variations in specimen structure. If figure 6 is inspected together with figure 2 then it is apparent that low temperature resistivity values follow the trend indicated by Deem et al. and Ermolaev, namely that resistivity increases to a maximum and then decreases. The data of the present work indicate that electrical resistivity decreases until 1250 K where the curve shows a kink but still decreases until 1400 K above which resistivity begins to increase steadily up to 1900 K. Above 1400 K, the resistivity results pertain to the beta phase. Below 1400 K however, a number of solid state reactions may be occurring, chief of which are the reactions whereby alpha prime decomposes to alpha + beta and, at high temperatures, alpha will decompose to beta. Additionally, beta is metastable at low temperatures and will itself decompose to alpha. Since these are kinetic processes affected by the heating rate, it is difficult to predict the behavior of this alloy unless further work is done involving the heating of alloys carefully heat treated to produce different structures.

No data have been found in the literature on the hemispherical total emittance of the alloy 90Ti-6Al-4V that correspond to the specimen conditions and the temperature range of the measurements of the present work. The value of the hemispherical total emittance for a similar alloy at the highest reported temperature (about 1200 K) in the literature [7] is 0.47 which corresponds to a specimen with a rough surface. This high value compared to 0.39 (the value of the present work) may be attributed primarily to the differences in the specimen surface conditions.

Measurements of the normal spectral emittance at 665 nm on several titanium alloy specimens under different conditions were reported in the literature [8] for temperatures up to about 1700 K. The reported results for the specimen and conditions that closely match those of the present work indicate a nearly linear variation of emittance with temperature in the range 1200 to 1700 K. Extrapolation of these results to the melting point of the alloy (1943 K) yields a value of 0.369 for the normal spectral emittance, which is about 7 percent lower than the value of 0.395 obtained in the present work. This difference may not be very significant due to the fact that specimens were different and the literature results were extrapolated to the melting point.

According to a compilation on melting points [9], the results, reported by different investigators, of the melting point of pure titanium are in the range 1933 to 1953 K. The value 1943 K obtained in the present work for the alloy is compatible with the above.

In the present experiments related to melting (true temperature obtained using tube-shape specimens and radiance temperature obtained using strip-shape specimens), it was not possible to follow the entire melting process because the specimen collapsed and opened the main electrical circuit prior to the completion of melting. However, good quality horizontal plateaus were obtained during the initial melting period which are more of a characteristic of pure metals.

## Figures and Tables

**Table 1:** Composition of the titanium alloy^a^

Material - Form	in wt. *%*			in ppm																			
	Ti	Al	V	B	Ca	Cr	Cu	Fe	Mg	Mn	Mo	Ni	Nb	Pb	Si	Sn	Ta	W	Zr	C	H	N	O
																							
Rod	89.2	6.1	4.2	10	50	15	30	1500	50	30	150	15	–	50	300	300	100	50	30	220	80	120	1700
Plate	89.2	6.1	4.2	30	150	30	80	1500	30	60	80	30	100	15	80	80	100	15	30	390	30	90	1500

a Information was furnished by Dr. S. R. Lyon of the U. S. Air Force Materials Laboratory.

**Table 2:** Specific heat capacity and electrical resistivity of the titanium alloy

*T* (K)	*C_p_* (J · g^−1^ · K^−1^)	*ρ* (*μ*Ω · cm)
		
1450	0.729	172.3
1500	.734	172.7
1550	.742	173.1
1600	.751	173.4
1650	.762	173.7
1700	.775	174.0
1750	.790	174.3
1800	.807	174.6
1850	.826	174.8
1900	.846	175.1

**Table 3:** Summary of measurements of the melting point of the titanium alloy

Specimen number	Number of temperatures at plateau	Melting point (K)	Standard deviation (K)
			
1	10	1942.7	0.4
2	23	1943.5	.6
3	25	1942.0	.3
4	15	1942.4	.2

**Table 4:** Summary of measurements of the radiance temperature (at 653 nm) of the titanium alloy during melting

Specimen number	Typical surface Roughness (*μ*m)	Number of temperatures at plateau	Radiance temperature (K)	Standard deviation (K)
				
1	0.4	27	1795.4	0.2
2	.5	56	1795.6	.5
3	.5	64	1795.2	.3
4	.4	36	1795.8	.4
5	.9	43	1795.9	.3

**Table 5:** Estimate of errors

Quantity	Imprecision^a^	Inaccuracy^b^
		
Specific heat capacity	0.7%	3%
Electrical resistivity	0.2%	1%
Hemispherical total emittance	2%	5%
Melting point	0.6 K	8 K
Radiance temperature (at melting point)	0.5 K	8 K
Normal spectral emittance (at melting point)	–	3%

a Imprecision refers to the standard deviation of a quantity as computed from the difference between measured value and that from the smooth function obtained by the least squares method.

b Inaccuracy refers to the estimated total error (random and systematic).

## Footnotes

1 Alloys have a melting range instead of a melting point, however for convenience, in the present work the solidus point of the alloy is referred to as the melting point.

2 Radiance temperature (sometimes referred to as brightness temperature) is the apparent temperature of the specimen surface as measured by the pyrometer.

3 Figures in brackets indicate the literature references at the end of this paper.

## 5. Appendix

**Table A-1:** Experimental results on specific heat capacity of the titanium alloy

Range	Temperature (K)	Specimen-1		Specimen-2		Specimen-3		Specimen-4	
		*C_p_* (J·g^−1^·K^−1^)	Δ*c_p_*^a^ (%)	*C_p_* (J·g^−1^·K^−1^)	Δ*c_p_*^a^ (%)	*C_p_* (J·g^−1^·K^−1^)	Δ*c_p_*^a^ (%)	*C_p_* (J·g^−1^·K^−1^)	Δ*c_p_*^a^ (%)
									
I	1450	0.7247	−0.58	0.7230	−0.81	0.7213	−1.05	0.7345	+0.76
	1500	.7332	−0.16	.7327	−0.23	.7305	−0.53	.7380	+0.49
	1550	.7431	+0.18	.7437	+0.26	.7414	−0.05	.7441	+0.31
	1600	.7550	+0.52	.7556	+0.60	.7544	+0.44	.7540	+0.39
	1650	.7693	+0.92	.7679	+0.74	.7700	+ 1.01	.7691	+0.90
									
II	1650	0.7595	−0.36	.7620	−0.03	.7619	−0.04	.7615	−0.09
	1700	.7710	−0.55	.7733	−0.25	.7747	−0.07	.7767	+0.19
	1750	.7862	−0.50	.7880	−0.27	.7902	+0.01	.7928	+0.34
	1800	.8059	−0.12	.8064	−0.06	.8091	+0.28	.8100	+0.39
	1850	.8311	+0.68	.8295	+0.48	.8320	+0.78	.8286	+0.38
									
III	1700	0.7775	+0.29	.7771	+ 0.24	.7820	+0.87	.7812	+0.77
	1750	.7820	−1.04	.7858	−0.55	.7836	−0.83	.7860	−0.52
	1800	.7952	−1.47	.7999	−0.87	.7948	−1.52	.7983	−1.07
	1850	.8182	−0.89	.8206	−0.60	.8175	−0.98	.8197	−0.71
	1900	.8538	+0.92	.8492	+ 0.44	.8557	+ 1.13	.8529	+0.81

a The quantity Δ*c_p_* is percentage deviation of the individual results from the smooth function represented by equation (1).

**Table A-2:** Experimental results on electrical resistivity of the titanium alloy

Range	Temperature (K)	Specimen-1		Specimen-2		Specimen-3		Specimen-4	
		*ρ* (*μ*Ω·cm)	Δ*ρ*^a^ (%)	*ρ* (*μ*Ω·cm)	Δ*ρ*^a^ (%)	*ρ* (*μ*Ω·cm)	Δ*ρ*^a^ (%)	*ρ* (*μ*Ω·cm)	Δ*ρ*^a^ (%)
									
I	1450	172.19	−0.08	172.80	+0.27	172.04	−0.17	172.31	−0.01
	1500	172.56	−0.09	173.21	+0.29	172.37	−0.20	172.59	−0.07
	1550	172.94	−0.08	173.57	+0.29	172.74	−0.19	173.02	−0.03
	1600	173.32	−0.05	173.90	+0.28	173.13	−0.16	173.47	+0.03
	1650	173.66	−0.04	174.22	+0.28	173.50	−0.14	173.77	+ 0.02
									
II	1650	173.75	+ 0.01	174.22	+0.28	173.41	−0.19	173.68	−0.03
	1700	174.09	+0.03	174.54	+0.29	173.72	−0.18	174.00	−0.02
	1750	174.41	+0.05	174.83	+0.30	174.03	−0.17	174.28	−0.02
	1800	174.70	+ 0.06	175.12	+0.30	174.32	−0.15	174.56	−0.02
	1850	175.00	+ 0.10	175.45	+0.35	174.56	−0.15	174.85	+0.01
									
III	1700	174.18	+0.08	174.18	+0.08	173.51	−0.30	173.72	−0.18
	1750	174.52	+ 0.11	174.51	+0.11	173.79	−0.31	174.02	−0.17
	1800	174.80	+ 0.12	174.81	+0.13	174.13	−0.26	174.29	−0.17
	1850	175.06	+ 0.13	175.07	+0.14	174.45	−0.22	174.54	−0.17
	1900	175.36	+0.17	175.30	+0.14	174.62	−0.25	174.77	−0.16

a The quantity Δ*ρ* is percentage deviation of the individual results from the smooth function represented by equation (2).

**Table A-3:** Experimental results on hemispherical total emittance of the titanium alloy

Specimen	*T* (K)	ϵ
		
1	1714	0.394
	1896	.388
		
2	1705	0.380
	1885	.376
		
3	1706	0.392
	1879	.392
		
4	1710	0.386
	1879	.380

## References

[1] Cezairliyan A (1971). Design and Operational Characteristics of a High-Speed (Millisecond) System for the Measurement of Thermophysical Properties at High Temperatures. J Res Nat Bur Stand (US).

[2] Cezairliyan A, Morse MS, Berman HA, Beckett CW (1970). High-Speed (Subsecond) Measurement of Heat Capacity, Electrical Resistivity, and Thermal Radiation Properties of Molybdenum in the Range 1900 to 2800 K. J Res Nat Bur Stand (US).

[3] (1969). International Practical Temperature Scale of 1968. Metrologia.

[4] Ziegler WT, Mullins JC (1961). Specific Heat of Titanium Alloys, Georgia Inst. Tech., Final Report.

[5] Deem HW, Wood WD, Lucks CF (1958). The Relationship Between Electrical and Thermal Conductivities of Titanium Alloys. Trans Met Soc AIME.

[6] Ermolaev BI (1974). Thermal Conductivity and Electrical Conductivity of Materials Based on Titanium and Its Alloys from 20–80 to 1000 K. Metal Science and Heat Treatment.

[7] de L’Estoile H, Rosenthal L (1958). Advisory Group for Aeronautical Research and Development, Paris, France.

[8] Betz HT, Olson OH, Schurin BD, Morris JC (1957). WADC-TR-56-222 (Part 2).

[9] Charlesworth JH (1970). Melting Points of Metallic Elements and Selected Compounds, Air Force Materials Laboratory Report.

